# Deciphering the Variants Located in the *MIR196A2*, *MIR146A*, and *MIR423* with Type-2 Diabetes Mellitus in Pakistani Population

**DOI:** 10.3390/genes12050664

**Published:** 2021-04-28

**Authors:** Muhammad Sohail Khan, Bashir Rahman, Taqweem Ul Haq, Fazal Jalil, Bilal Muhammad Khan, Saleh N. Maodaa, Saleh A. Al-Farraj, Hamed A. El-Serehy, Aftab Ali Shah

**Affiliations:** 1 Department of Biotechnology, University of Malakand, Chakdara 18800, Pakistan; sohailkhanct45@gmail.com (M.S.K.); bashirrahman697@gmail.com (B.R.); taqweembiotech@yahoo.com (T.U.H.); 2 Department of Biotechnology, Abdul Wali Khan University Mardan (AWKUM), Mardan 23200, Pakistan; fazaljalil@awkum.edu.pk; 3 University Institute of Biochemistry and Biotechnology, Pir Mehr Ali Shah Arid Agriculture University, Rawalpindi 46300, Pakistan; dr.bilal@uaar.edu.pk; 4 National Center of Industrial Biotechnology, Pir Mehr Ali Shah Arid Agriculture University, Rawalpindi 46300, Pakistan; 5 Department of Zoology, College of Science, King Saud University, Riyadh l1451, Saudi Arabia; smaodaa@ksu.edu.sa (S.N.M.); alfarraj@ksu.edu.sa (S.A.A.-F.); helserehy@ksu.edu.sa (H.A.E.-S.)

**Keywords:** T2DM, *MIR196A2*, *MIR146A*, *MIR423*, MicroRNA, single-nucleotide polymorphisms

## Abstract

MicroRNAs (miRNAs) are small non-coding RNA molecules that control the post-transcriptional gene expression. They play a pivotal role in the regulation of important physiological processes. Variations in miRNA genes coding for mature miRNA sequences have been implicated in several diseases. However, the association of variants in miRNAs genes with Type 2 Diabetes Mellitus (T2DM) in the Pakistani population is rarely reported. Therefore, the current study was designed to investigate the association of rs11614913 T/C (*MIR196A2*), rs2910164 G/C (*MIR146A*), and rs6505162 C/A (*MIR423*) in clinicopathological proven T2DM patients and gender-matched healthy controls. The tetra-primer amplification refractory mutation system-polymerase chain (ARMS-PCR) reaction method was used to determine the genotypes and to establish the association of each variant with T2DM through inherited models. In conclusion, the present study showed that variants rs11614913 T/C and rs2910164 G/C were linked with the risk of T2DM. The data suggested that rs11614913 T/C and rs2910164 G/C could be considered as novel risk factors in the pathogenesis of T2DM in the Pakistani population.

## 1. Introduction

Diabetes Mellitus (DM) is a group of metabolic disorders categorized by high glucose levels in the blood. DM is characterized by hyperglycemia due to insufficient production of insulin or insulin resistance or both [1]. The World Health Organization (WHO) further classified DM into four sub-groups: T1DM, T2DM, Gestational Diabetes Mellitus (GDM), and Maturity-onset diabetes of youth (MODY). In the genetic factor, both protein-coding genes and non-coding genes are involved in the pathophysiology of DM. According to the international diabetic survey, in 2019, about 19.4 million people were diagnosed with diabetes in Pakistan [2,3]. It is one of the leading causes of death in Pakistan. The estimated incidence of diabetes is higher in an urban area (28.3%) than in a rural area (25%). In Pakistan, the risk factors for DM are family history, hypertension, obesity, dyslipidemia, and age. Due to its increasing rate, DM is the third most common cause of mortality worldwide. In the Pakistan diabetic cohort, the reported mortality rate was 15.9%, with higher mortality being reported in those aged 65 years.

In higher organisms, only a small percentage of the whole genetic transcripts (less than 3%) can encode proteins, whereas much of the rest of the genome produce non-coding RNAs such as microRNAs (miRNAs), an abundant class of small (~22 nucleotides) regulatory RNAs. These miRNAs regulate the post-transcriptional expression of genes. The miRNA biogenesis involves the processing of long primary transcripts into short precursors, termed pre-miRNAs, which form stable RNA hairpin structures. These pre-miRNA hairpins are subsequently further processed into the mature miRNA form, single-strand RNAs ~22 in length [4]. A total of 1917 precursor miRNAs are expressed that lead to the production of 2654 mature miRNAs in humans [5]. These expressed mature miRNAs regulate virtually every aspect of the living cell [6]. Several risk factors of Type 2 Diabetes Mellitus (T2DM) have been studied. However, the role of non-coding genome in the pathophysiology of T2DM is at infancy.

Single-nucleotide polymorphisms (SNPs) are a common genetic variation in the genome. It is already known that SNPs occur more commonly in the non-coding part of the genome [7]. It has been shown that SNPs in miRNAs may influence both their expression and function, leading to human disease susceptibility [8]. It is therefore not surprising that the distraction of miRNA function leads to many human diseases [9]. SNPs located in the candidate miRNA genes can affect the expression of corresponding targets involved in the pathophysiology of T2DM. MiRNAs seem to play a role in the development of pancreatic islets and the differentiation of insulin-producing cells [10]. For instance, mir-375 is involved in insulin secretion by interaction with the myotrophin gene and the development of islets. Polymorphisms in miRNA genes may influence the miRNA maturation and thereby modulate miRNA expression, leading to dysregulation of target mRNA. The SNP in *MIR146A* (rs2910164 G/C) is a G to C transition at the 60th nucleotide of the gene thereby leading to reduced expression of mature hsa-miR-146a. Previous studies have shown that rs2910164 G/C polymorphism is associated with several diseases [11,12,13]. The rs2910164 G/C increased the risk of T2DM in the Chinese and Caucasian populations [14,15]. It has been shown that hsa-miR-146a reduces the activity of NF-kappa B [16], leading to complications of diabetes [17,18]. The SNP (rs2910164 G/C) is located within the stem-loop of hsa-miR-146a. The polymorphism rs6505162 C/A has been reported to promote the expression of mature mir-423 [19]. This polymorphism is linked with various diseases such as esophageal cancer [20] and breast cancer [21]. Previous studies have shown the association between *MIR196a2* rs11614913 TC polymorphism and Kawasaki disease susceptibility in southern Chinese children [22], lung cancer [23,24], gastric cancer [25], colorectal cancer [26], spontaneous abortion, and breast cancer [27]. It is already known that SNP rs11614913 is in *MIR196A* and is located in chromosome 17 between *HOX* genes. It also interacts with *HOX* genes [28,29]. The previous study has shown that *HOX* genes play a pivotal role in the initiation of fetal organs, including the pancreas. Furthermore, the mature miRNAs of *MIR196A* can activate the AKT signaling pathway. This biology is involved in the development and treatment of type 2 diabetes [30,31] revealing the key role of miR-196 in the pathogenesis of T2DM.

In the present study, we performed a case-control study in the Pakistani population to investigate whether the SNPs (rs11614913 T/C, rs2910164 G/C, and rs6505162 C/A) in *MIR196A2, MIR146A,* and *MIR423* are involved in the pathogenesis of T2DM.

## 2. Materials and Methods

### 2.1. Clinical Data, Blood Sample Collection, and Ethical Approval

The present study was conducted according to the Helsinki declaration [32]. Basic information about each patient and healthy control, e.g., gender, height, age, body mass index (BMI), low-density lipoprotein (LDL), high-density lipoprotein (HDL), random blood sugar (RBS), cholesterol level, and family history were recorded (Table 1). A total of 346 cases and 333 healthy individuals were included in the current study. About 5 ml whole blood was collected from each individual and genomic DNA was extracted using the standard method [33].

### 2.2. Selection of SNPs

The information about the genomic locations of currently 1917 precursors and 2654 human mature miRNAs are available in the miRbase (http://www.mirbase.org). Relevant information about the studied SNPs was retrieved from miRbase. The information regarding chromosomal locations and alleles information of rs11614913 T/C, rs6505162 C/A, and rs2910164 G/C were downloaded from the dbSNP. An online tool SNPinfo Web Server (http://snpinfo.niehs.nih.gov/snpinfo/snpfunc.html) was used to predict the functional consequences of these studied SNPs in the secondary structure of miRNAs.

### 2.3. Genotyping Assay

Tetra-primer amplification refractory mutation system–PCR (ARMS–PCR) is a simple and cost-effective SNP genotyping technique. This allele-specific method is based on the utilization of allele-specific primers containing a mismatch in their 3′ terminus, making this primer specific to only one allele of the SNP and refractory to the other allele. In the tetra-primer, the tetra-primer ARMS–PCR uses four primers in a single PCR to determine the genotype. At the beginning of the reaction, two non-allele-specific primers amplify the region that comprises the SNP. They are named outer primers, then. As the outer primer fragment is produced, it serves as a template to the two allele-specific primers (inner primers) which will produce the allele-specific fragments. In this way, they reduce the chances of false-positive results [34,35,36]. The online tools primer 1 (http://primer1.soton.ac.uk/primer1.html) was used for designing the primers for the selected SNPs. The T-ARMS PCR was used for genotyping of rs2910164 G/C, rs6505162 C/A, and rs11614913 T/C polymorphisms using two inner allele-specific primers (FI and RI) and two outer primers (FO and RO) for each SNP, as shown in Table 2. as shown previously [37,38].

### 2.4. Functional Prediction Analysis and Landscaping of the Mutated miRNA Sequences

The RNAfold (http://rna.tbi.univie.ac.at/cgi-bin/RNAWebSuite/RNAfold.cgi) is an online tool that can be used to predict the secondary structure of RNA sequence through the calculation of the thermodynamic ensemble of RNA structures, minimum free energy, and the centroid structure. This tool was used to predict the effects of rs11614913 T/C, rs2910164 G/C, and rs6505162 C/A on the secondary structure of their corresponding mature miRNAs.

### 2.5. Statistical Analysis

The Hardy–Weinberg equilibrium was applied to analyze the observed and expected genotype frequencies for both cases and controls. All four inheritance models (codominant, dominant, recessive, and additive) were used to calculate the allelic and genotypic frequencies of these studied SNPs. The odds ratio (OR) at 95% confidence intervals (CIs) was also calculated (Table 3).

## 3. Results

### 3.1. Association of rs11614913 T/C, rs6505162 C/A, and rs2910164 G/C with Increased Risk of Type 2 Diabetes *Mellitus*

The distribution of genotypes and allele frequencies for the variants (rs11614913 T/C, rs6505162 C/A, and rs2910164 G/C,) in both cases and healthy controls are shown in Table 3. The genotype frequencies were in Hardy–Weinberg Equilibrium for all three variants. The error rate in the genotypes was estimated by re-genotyping of 10% of study subjects. Each variant was statistically tested under co-dominant, dominant, recessive, and additive genetic models. Statistically, a strong association was found between the C allele of rs11614913 T/C (*MIR196A2)* and increased risk of T2DM (*p*_co-dom_ < 0.0001, *p*_dom_ < 0.0001, *p*_rec_ = 0.0127, and *p*_add_ < 0.0001, respectively). Similarly, the G allele of rs2910164 G/C (*MIR146A*) was an indicator of T2DM risk (*p*_co-dom_ = 0.0001, *p*_dom_ = 0.0001, *p*_rec_ = 0.018 and *p*_add_ = 0.0001). On the other hand, no association was established between rs6505162 C/A (*MIR423*) and an increased risk of T2DM.

### 3.2. Effect of SNPs on the Secondary Structure of miRNAs

**Figure 1:**
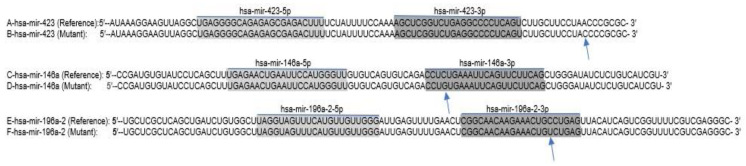
The primary sequences of hsa-miR-423 (reference: **A**), (mutant: **B**), hsa-miR-146a (reference: **C**), (mutant: **D**), hsa-miR-196A2 (reference: **E**), (mutant: **F**).

The studied SNPs had a detrimental effect on the predicted secondary structure of the pre-miRNAs (Figure 1). The SNP rs11614913 T/C is located near the primary cleavage site necessary to generate pre-hsa-miR-196a2. Thus, this mutation may impact the biogenesis of mature miR196a. The predicted minimum free energy by the C allele of rs11614913 T/C was −50.30 kcal/mol, the free energy of the thermodynamic ensemble was −52.02 kcal/mol and the minimum free energy (MFE) centroid of the secondary structure in dot-bracket notation was −49.90 kcal/mol, whereas the minimum free energy, free energy of the thermodynamic ensemble, and the minimum free energy centroid of the secondary structure by the T allele of rs11614913 T/C were −44.70 kcal/mol, 46.52 kcal/mol, and −44.30 kcal/mol, respectively.

## 4. Discussion

SNP is one of the most common genetic variations in a genome [39]. Previous studies have revealed that SNPs within the miRNAs region are involved in complex traits and diseases including T2DM [40,41,42]. However so far, the SNPs (rs11614913 T/C, rs2910164 G/C, and rs6505162 C/A) within *MIR196A2, MIR146A,* and *MIR423* have not been screened for association with T2DM in the Pakistani population. The study aimed to explore the association of rs11614913 T/C, rs2910164 G/C, and rs6505162 C/A in *MIR196A2, MIR146A,* and *MIR423*, with T2DM in the Pakistani population. A few SNPs are supposed in pre-miRNA hairpin districts, one of these SNPs, rs11614913 T/C, is found in the 3p arm of *MIR196A2* [43]. For each variant, the total number of study subjects were genotypes but the individuals with undetermined-genotype calls were removed as a step of data filtration. The individuals with confirmed genotype calls were included in the statistical analysis for the association’s study.

A previous study has shown that miR-196a rs11614913 T/C is linked with T2DM [29]. Downregulation of its mature miRNA may play a pivotal role in T1DM [44]. In the current study, we found that the genotype TT (n = 76/22.4%) of rs11614913 T/C was more frequent in cases as compared to controls (n = 33/13.98%). On the other hand, the genotype CC was more common in the control group (n = 130/38.46%) than patients (n = 84/35.59%). In patients, the heterozygote CT was more common (n = 178/52.66%) than the control group (73/21.59%). Statistical analysis revealed that the CC genotype reduces the cause of T2DM with an odds ratio of 0.26 (CI = 0.1889–0.3849), while in the recessive model the genotype TT increased the risk of T2DM with an odds ratio of 1.784 (CI = 0.1889–0.3849). Comparison of alleles C and T showed a reduced risk for the C allele (OR = 0.4377). Following our results, in a study carried out in Cairo, Egypt showed that the frequency of TT genotype was more common in patients than the healthy individuals. In support of our study, this study also found a significantly higher frequency of the T allele in patients than in controls (41.7% vs. 31.3%), while the C allele was more frequent in controls. The study stated that the association of the TT genotype with T1DM remained significant. The study also reported decreased relative expression of miR-196a2 in patients compared to controls [44]. In contrast to our studies, some researchers have reported a higher frequency of T alleles in patients than controls and the T allele has been reported to decrease the risk of T2DM. Previous studies have confirmed that the CC genotype (C allele) is linked with various types of cancers. A study indicated that individuals with genotype CC are at lower risk of BC as compared with controls, whereas those with CT genotype are at higher risk of breast cancer. Previously, it was found that the C allele increased the risk of gastric cancer and colorectal cancer [45]. It was found that the C allele of rs11614913 T/C upregulated the expression of miR-196a2 up to 9-folds in cells transfected with miR-196a2-C but increased only by 4.5-fold with the miR-196a2T allele. Increased expression of miR-196a2 was associated with the C allele. It was noted that miR-196a2 rs11614913 T/C not only affects the expression level of mature miR196a but also has a phenotypic consequence on its target gene expression. The results demonstrated that a total of 684 mutated miRNAs of *MIR196A* were found following the introduction of miR-196a-C, while less than 342 miRNAs were changed after the introduction of miR-196a-T [46]. Furthermore, it was assumed that the T allele may be linked with decreased expression of miR-196a2. Another interesting fact is that part of the inflammatory response mounting in T2D is related to autoimmunity. The miR-196a2 plays a crucial role in the regulation of immune responses through the annexin A1 and transforming growth factor signaling pathways [47,48]. Downregulation of miR-196a2 resulted in the overexpression of *MAPK1* gene leading to the T cell receptor signaling pathways [49]. Furthermore, it was also investigated that miR-196a2 is involved in the insulin signaling pathways.

A recent study suggests that miR-146a plays a crucial role in the prognosis of DM by contributing to the metabolism, proliferation, and death of β-cell. It can be detected in serum, T cells, and α- and β-cells of patients with DM, supposing that the miR-146a is a therapeutic target and potential biomarker [50,51]. A previous study demonstrated that rs2910164 G/C leads to an unstable pre-miR-146a structure. The current study was carried out in 94 patients and 69 healthy individuals. By applying a co-dominant statistical model GG vs. GC vs. CC, a significant difference in genotype frequencies was observed (*p*-value = 0.0001). The genotype GG was more frequent in control group (62.3%) as compared to diabetes patients (13.8%), whereas the genotype GC was more common in patients (52.1%) as compared to control (23.1%). The homozygote CC was also more common in patients (34.0%) than in control (14.5%). The genotype GG reduced the risk of diabetes with an odds ratio of 0.2439 (CI = 0.1524–0.3902), whereas the recessive model showed an increased risk for the genotype CC OR = 2.123 (CI = 1.118–4.033). Comparison of the two alleles (G and C) showed a reduced risk for G allele 0.2439 (CI = 0.1524–0.3902). Thus, it can be concluded that the C allele of rs2910164 G/C in miR-146a increases the risk of T2DM. Similarly, in another study, it was found that the C allele of rs2910164 G/C in miR-146a may increase the risk of developing T2DM (*p* < 0.001, OR = 1.459; 95% CI: 1.244–1.712) in the Han Chinese population. They also reported a higher frequency of CC genotype (49%) in patients as compared to control (38%), while the genotype GG was more common in the control group (17%) as compared to patients (10%). The previous study has also shown that the two common variants: rs2910164 G/C, rs11614913 T/C were associated with the development of several diseases including type 2 diabetes mellitus (T2DM) [52,53,54,55,56].

The SNP rs6505162 C/A is situated in the pre-miRNA sequence of hsa-mir-423 (MI0001445) that encodes two mature miRNAs (hsa-miR-423-3p and hsa-miR-423-5p). Hypothetically, the SNP in the pre-miRNA sequence can alter the miRNA processing and expression. This alteration in the pre-miRNA may lead to the dysfunction of crucial biological pathways [57].

The current study included 48 patients and 44 control individuals. Statistical analysis did not reveal any significance in genotype frequencies. The frequencies of genotypes AA, AC and CC were 25%, 45.8% and 29.1%, respectively, while in control these frequencies were AA = 20.5%, AC = 54.5%, and CC = 25%. Other studies have identified the association of rs6505162 C/A with the risk of Esophageal Squamous Cell Carcinoma [58]. Although rs6505162 C/A is not situated in the seed region of the mature miRNAs, it has been proposed that rs6505162 C/A can alter not only the mature miR-423 expression [59] (Zhao et al., 2015), but it may also modulate its targeting efficiency [19]. Other studies have shown that the CA/AA genotypes of rs6505162 C/A could reduce the occurrence of artery aneurysms [59]. The rs6505162 C/A is reported to have a role in different diseases including Type 2 Diabetes. However, in the current study, we did not find any association of the SNP rs6505162 C/A with diabetic patients from Pakistan.

This study is purely based on genotyping of selected SNPs in *MIR196A2*, *MIR146A*, and *MIR423* in patients with T2DM as compared to healthy controls. It does not show the altered expression level of matured miRNAs encoded by these studied genes. Future study should be performed to validate few selected targets by luciferase assay and to investigate the effect of these mutations in miRNAs modulating gene expression of the targets.

## Figures and Tables

**Table 1:** Upper and lower ranges and mean values of different variables in Type 2 Diabetes Mellitus (T2DM) patients and healthy controls.

Category	Age (Years) Mean (Range)	BMI (kg/m^2^) Mean (Range)	RBS (mg/dL) Mean (Range)
T2DM (M)	57.4 (32–100)	23.4 (13.5–39.8)	245 (135–510)
T2DM (F)	55.3 (29–91)	25.2 (12.3–37.3)	244.2 (144–510)
CONTROL	44 (10–100)	19.6 (14.5–33.7)	138.9 (70–168)

**Table 2:** Tetra-primer amplification refractory mutation system–PCR (T-ARMS PCR) primers were used for the genotyping of rs2910164, rs11614913, and rs6505162.

SNP Name	Primer	Primer Sequences	PCR Product Size
rs2910164	FI (C)	5-ATGGGTTGTGTCAGTGTCAGACGTC-3	169 bp
	RI (G)	5-GATATCCCAGCTGAAGAACTGAsATTTGAC-3	249 bp
	FO	5-GGCCTGGTCTCCTCCAGATGTTTAT-3	364 bp
	RO	5-ATACCTTCAGAGCCTGAGACTCTGCC-3	
rs11614913	FI (T)	5-AGTTTTGAACTCGGCAACAAGAAACGGT-3	199 bp
	RI (C)	5-GACGAAAACCGACTGATGTAACTCCGG-3	153 bp
	FO	5-ACCCCCTTCCCTTCTCCTCCAGATAGAT-3	297 bp
	RO	5-AAAGCAGGGTTCTCCAGACTTGTTCTGC-3	
rs6505162	FI (C)	5-GCCCCTCAGTCTTGCTTCCCAC-3	199 bp
	RI (A)	5-GGGGAGAAACTCAAGCGCGAGT-3	292 bp
	FO	5-GGGATGAGAAACTACGGCGACTGTATCT-3	447 bp
	RO	5-TATGCCTACCCTTTTTCTGTGGCTTCTC-3	

**Table 3:** Statistical analysis of rs11614913C/T, rs2910164G/C and rs6505162A/C using the co-dominant, dominant, recessive, and additive models.

Name of SNP/Gene	Statistical Models	Genotypes	Cases (n = 346)	Controls (n = 333)	Odds Ratiο (95% Cl)	χ^2^-Value, df	*p*-Value
**rs11614913/*MIR196A2***	Co-Dominant	CC CT TT	84 178 76	130 73 33	---	54.4, 2	<0.0001
	Dominant	CC CT + TT	84 254	130 106	0.2697 (0.1889–0.3849)		<0.0001
	Recessive	TT CT + CC	76 262	33 203	1.784 (1.140–2.793)		0.012
	Additive	C T	346 330	333 139	0.4377 (0.3412–0.5613)		<0.0001
**rs2910164/*MIR146A***	Co-Dominant	GG GC CC	13 49 32	43 16 10	---	47.1, 2	0.0001
	Dominant	GG GC + CC	13 91	43 26	0.2439 0.1524–0.3902		0.0001
	Recessive	CC GC + TT	32 72	10 59	2.123 (1.118–4.033)		0.01
	Additive	G C	85 123	102 34	0.2439 (0.1524–0.3902)		0.0001
**rs6505162/*MIR423***	Co-Dominant	AA AC CC	12 22 14	9 24 11	---	0.7029, 2	0.70
	Dominant	AA AC + CC	9 46	22 36	1.296 (0.4856 to 3.460)		0.62
	Recessive	CC AC + AA	22 33	2 34	1.235 (0.4904–3.112)		0.81
	Additive	A C	46 50	42 46	1.008 (0.5646–1.798)		1
